# In Tribute to Joep Lange

**DOI:** 10.1186/s12977-014-0082-z

**Published:** 2014-09-20

**Authors:** Catherine A Hankins, Mark A Wainberg, Robin A Weiss

**Affiliations:** Department of Global Health, Academic Medical Centre, University of Amsterdam and Amsterdam Institute for Global Health and Development, Amsterdam, The Netherlands; McGill AIDS Centre, Lady Davis Institute for Medical Research, Jewish General Hospital, 3999 Côte-Sainte-Catherine Road, F-328, Montréal, QC H3T 1E2 Canada; London School of Hygiene and Tropical Medicine, London, UK

We commemorate the sad loss of a true leader in retroviral disease, Professor Joep Lange, former President of the International AIDS Society. Our friend and colleague Joep perished on July 17, 2014 together with his beloved partner Jacqueline van Tongeren, while flying from Amsterdam to the International AIDS Conference in Melbourne aboard Malaysian Airlines flight 17. Joep contributed in compelling fashion to much of the progress accomplished thus far in combatting the HIV epidemic. He lost his life together with Jacqueline and four other individuals on their way to the conference, shot down by killers whose own families and friends might have benefited in the past and may benefit in the future from Joep’s many contributions to our field.

Joep was pivotally involved from the earliest days of anti-retroviral therapy, helping devise the important strategies for combatting HIV infection that continue to be employed today. Together with Jaap Goudsmit, Frank Miedema, and Roel Coutinho, he played a key role in establishing the multidisciplinary group of investigators in Amsterdam that pioneered much of our understanding today of HIV and its disease course. They established well-characterised cohorts of HIV-positive people and those at higher risk of HIV exposure, sought to understand transmission dynamics, and helped elucidate HIV pathogenesis, progression to AIDS, and the benefits of early treatment. From the mid-1980s onwards, the Dutch group made immense contributions to the field and it did not take long for Joep to begin to translate the insights gained in Amsterdam into strategies to ameliorate the burden of AIDS in Africa.

He began his career at the University of Amsterdam, receiving his MD in 1981 and PhD in 1987. From 1992 to 1995, he was Chief of Clinical Research and Drug Development at the Global Programme on AIDS at the World Health Organization in Geneva. Joep played a key role at WHO during the time that the spread of HIV was proceeding virtually unabated and when very few antiretroviral drugs were available. He advocated passionately for and was involved in the conduct of some of the earliest clinical trials that embraced the simultaneous use of three drugs to combat viral replication. Indeed, he was among the first to understand that such a strategy was mandated by the ability of HIV to mutate rapidly, both in the absence and presence of drug pressure. Throughout his career, Joep maintained a keen interest in questions relating to viral pathogenesis and conducted early work on this topic. His PhD thesis at the University of Amsterdam was entitled “Serological markers in HIV infection” and Joep was among the first to demonstrate that high levels of viral p24 protein in the circulation were a key prognostic indicator of disease progression.

Joep was a strong advocate for people living with HIV throughout the world, arguing repeatedly that they should have access to excellent antiretroviral drugs regardless of where they lived. When confronted with potential problems relating to drug access, he retorted: “If we can get cold Coca-Cola and beer to every remote corner of Africa, it should not be impossible to do the same with drugs”.

In recent years, Joep took a strong interest in the possibility of achieving HIV eradication from the body i.e. a cure for HIV infection. He keenly immersed himself in arguments as to the feasibility of approaches that might lead to an end to HIV transmission and his sharp mind was second to none in regard to how best to use all of our available tools toward this goal. Indeed, Joep was one of the first scientists to publish on the topic of cellular reservoirs of HIV and on HIV persistence. He was also among the first to understand that antiretroviral drugs might be used not only to treat individuals with HIV but also as agents to protect people from acquiring HIV infection, concepts that have now become firmly established within the field. He felt so strongly about HIV pre-exposure prophylaxis that he was dismayed when early trials encountered controversy. Joep was directly involved in studies on the prevention of HIV transmission from mothers with HIV infection to their offspring through the use of antiretroviral agents.

Joep played a key role in many international studies, beginning with efforts to deal with the HIV epidemic in Thailand together with colleagues in that country. He founded an organization termed PharmAccess aimed at providing affordable HIV care in Africa. Joep lectured throughout the world and participated on a regular basis in mentorship activities in The Netherlands and internationally. He trained numerous clinicians from low- and middle-income countries so that the benefits of antiretroviral therapy might become available to all.

Joep was the elected President of the International AIDS Society between 2002 and 2004, a role in which he proved to be extremely effective. Not only did he serve with devotion and commitment, but he travelled tirelessly and played a pivotal role in the organization of several international conferences, including the HIV Pathogenesis Conference in Paris in 2003 and the International AIDS Conference in Bangkok in 2004. He fought effectively for scholarship programs to be established at such conferences in order that individuals from developing countries might be able to participate and present their data. He was also insistent that capacity building programs become entrenched within the International AIDS Society (IAS) in further fulfillment of that organization’s mission. He established the IAS Industry Liaison Forum that brings together scientific industry representatives and experienced investigators from low- and middle-income countries, as well as high-income countries, to address key issues in HIV research in resource-limited settings, including considerations of post-trial care provision.

Joep’s partner Jacqueline worked as head nurse of the AIDS ward in Amsterdam before joining Joep as HIV research coordinator in 1990. She played a key role in clinical trial conduct and in international HIV educational efforts. She helped to guide Joep in his belief that the establishment of partnerships with academic scientists in developing country settings represented the best way to ensure that progress would be accomplished on an international basis. She took immense pleasure in organising the INTEREST Workshop, a stellar scientific conference held each year in Africa to profile the best in African HIV science. In truth, Jacqueline also helped to round out some of Joep’s rougher edges. Like many of those with a burning mission to help the world’s least fortunate people, he occasionally became exasperated by opinions that he considered to be foolish. Jacqueline made him more willing to listen to other points of view.

It is ironic that two giants in the HIV field have now perished in airplane crashes, the other, of course, being Jonathan Mann who died with 228 others on the Swiss Air Flight that crashed into the Atlantic ocean off the coast of Nova Scotia in 1998. The difference is that Jonathan’s demise was due to an aircraft accident while Joep’s death, together with those of 297 other victims aboard Malaysian Airlines flight 17, is considered to be an assassination.

In summary, Joep was a profound thinker who helped to change the world in regard to the diagnosis, treatment, and prevention of HIV disease. He exemplified the best to be expected in an academic physician, i.e. a sense of compassion, brilliance of leadership, and abhorrence of stigma for whatever reason. Possessing a burning desire to understand all aspects of the HIV epidemic, he became a knowledgeable and reliable commentator in areas such as epidemiology and pathogenesis that were outside his original clinical expertise.

Although we have lost a friend and a leader, we are confident that his memory will live on through activities that will be established throughout the world in his name. These may include Joep Lange memorial lectureships and scholarship and mentorship programmes dedicated to his memory that will assist low- and middle-income scientists and clinicians to obtain training and to participate actively in future conferences in our field.

